# Nanoliposome-Encapsulated Semiconductor Particles and Arsenic Trioxide Synergistically Enhance Chemo-Photothermal Therapy for Lung Cancer

**DOI:** 10.32604/or.2026.074880

**Published:** 2026-03-23

**Authors:** Chang He, An Wang, Youbo Wang, Qinyun Ma, Xiaofeng Chen

**Affiliations:** Department of Thoracic Surgery, Huashan Hospital, Fudan University, Shanghai, China

**Keywords:** Arsenic trioxide (ATO), poly(cyclopentadithiophene-alt-benzothiadiazole) (PCPDTBT), lung cancer, liposomes, tumor metastasis

## Abstract

**Objectives:**

Combined chemotherapy and photothermal therapy (PTT) represents a promising approach for enhancing cancer treatment efficacy. This study aimed to develop arsenic trioxide (ATO) and poly(cyclopentadithiophene-alt-benzothiadiazole) (PCPDTBT)-loaded nanoparticles (ATO/PCPDTBT@NPs) to evaluate their synergistic efficacy in inhibiting lung cancer growth and metastasis.

**Methods:**

Nanovesicles were synthesized via a streamlined protocol and subjected to 808 nm NIR irradiation to assess their photothermal conversion capabilities. The therapeutic efficacy was evaluated *in vitro* using A549 lung carcinoma cells to assess apoptosis, invasion, and migration, and *in vivo* to monitor tumor volume reduction.

**Results:**

The nanoparticles exhibited excellent hemocompatibility and low cytotoxicity while demonstrating robust photothermal conversion, inducing a rapid 20.8°C temperature rise within five minutes. *In vitro*, ATO enhanced apoptotic pathways and suppressed metastasis, while the combination therapy significantly reduced cell viability (OD: 0.23 vs. 0.62 in controls) and migration (13.6% vs. 74.9%), outperforming monotherapies. *In vivo*, the chemo-photothermal treatment reduced tumor volumes by 2.5- and 3-fold compared to ATO or PTT alone, confirming superior antitumor effects.

**Conclusions:**

These findings highlight the dual-action ATO/PCPDTBT@NPs nanoplatform as a potential multifaceted strategy for effective tumor suppression and metastasis inhibition.

## 1 Introduction

Lung cancer [1] is the leading cause of cancer-related deaths in men and the second most common cause of death in women (after breast cancer). In China, the incidence of lung cancer accounts for approximately one-third of global cases [2], with a continuous upward trend. Non-small cell lung cancer (NSCLC) is the most common type of lung cancer [3], representing 85% of all cases, mainly including adenocarcinoma and squamous cell carcinoma. Moreover, the survival rate of patients after treatment is only 10%–30% [4], with high rates of recurrence and metastasis. Currently, mature treatment methods for NSCLC patients include traditional surgery, chemotherapy, radiotherapy, etc. [5]. Among them, chemotherapy aims to eliminate tumor cells and metastatic malignant cells, but due to issues such as low selectivity of chemotherapy drugs for tumor cells and nonspecific delivery, there are problems such as insufficient drug dosage to kill tumor cells, adverse reactions, and the development of drug resistance by malignant tumor cells after exposure to chemotherapy drugs, leading to unsatisfactory chemotherapy outcomes [6].

Photothermal therapy (PTT) utilizes the near-infrared (NIR) laser for thermal ablation of malignant tumors [7]. It is reported that NIR has strong tissue penetration and can be converted into high thermal energy upon irradiation to destroy tumor cell membranes, cytoskeleton, and inhibit DNA synthesis [8], presenting incomparable advantages compared to traditional treatment methods [9]. PTT has several important advantages compared with other tumor treatment methods, especially suitable for superficial skin tissues [10]. Firstly, it has high local specificity, limiting therapeutic effects to specific locations through direct cytotoxicity. Secondly, PTT is minimally invasive and does not require general anesthesia [11]. Additionally, PTT combines local tumor control with overcoming immune suppression of remote diseases, thereby overcoming traditional resistance mechanisms and reducing side effects [12]. Finally, PTT can also be used as a fluorescent probe for photodynamic diagnosis, aiding in the precise localization of tumor margins, which is crucial for successful surgical resection [13]. Therefore, PTT is considered a powerful tool for tumor treatment and bioimaging [14,15].

Recently, semiconductor polymer nanoparticles have demonstrated significant potential in phototherapy diagnostics due to their high absorption coefficients, good photostability, and biocompatibility [16]. This design not only exhibits excellent therapeutic effects but also enables *in vivo* tumor imaging, providing new insights and methods for tumor treatment and diagnosis. While metal oxide-based nanoparticles have demonstrated promise in cancer therapy, their clinical translation faces inherent limitations. These inorganic systems typically exhibit restricted NIR absorption bandwidths (λ < 800 nm) and suboptimal photothermal conversion efficiencies (<35%), often requiring hazardous high-power irradiation. Notably, the polymer backbone of poly(cyclopentadithiophene-alt-benzothiadiazole) (PCPDTBT) demonstrates superior photostability compared to metal oxide quantum dots prone to photobleaching [17]. PCPDTBT is a donor-acceptor (D-A) type conjugated copolymer consisting of alternating cyclopentadithiophene (CPDT) donor units and benzothiadiazole acceptor moieties. The CPDT unit is functionalized with 2-ethylhexyl side chains to enhance solubility and processability, while the BT unit provides strong electron-withdrawing characteristics, facilitating intramolecular charge transfer. Our work strategically combines these merits by engineering PCPDTBT-based nanovesicles co-loaded with arsenic trioxide (ATO), addressing three critical gaps in prior studies: (a) simultaneous NIR-triggered hyperthermia and controlled drug release, (b) elimination of transition metal toxicity through all-organic photothermal cores, and (c) synergistic enhancement of apoptosis via thermal-potentiated chemotherapy [18].

Arsenic trioxide (ATO) [19], the main component of the traditional Chinese medicine arsenic, has a long history of medicinal use in China. In modern times, ATO was first used by scholars for the treatment of acute promyelocytic leukemia, demonstrating high efficacy and safety in antitumor activity [20]. With ongoing research, ATO has also shown significant potential in tumor-related treatments. Literature reports indicate that ATO can disrupt intracellular redox homeostasis by increasing reactive oxygen species (ROS) levels and decreasing glutathione (GSH), thereby inducing apoptosis via the intrinsic pathway [21]. ATO also induces apoptosis by modulating Bcl-2 family proteins. Moreover, recent studies have found that ATO has potential in regulating the immune system [22]. This suggests that ATO has promising applications in tumor treatment.

This study aimed to develop and evaluate nanovesicles co-loaded with arsenic trioxide (ATO) and the semiconducting polymer poly(cyclopentadithiophene-alt-benzothiadiazole) (PCPDTBT) (ATO/PCPDTBT@NPs) as a dual-function platform for synergistic chemo-photothermal therapy of lung cancer. We hypothesized that ATO/PCPDTBT@NPs, serving simultaneously as drug carriers and photothermal agents, would enhance antitumor efficacy and reduce systemic toxicity compared with chemotherapy or photothermal therapy alone. The resulting ATO/PCPDTBT@NPs demonstrated excellent hemocompatibility, minimal cytotoxicity, and highly efficient NIR-triggered photothermal ablation (PTA), and the combined chemo-photothermal treatment significantly improved therapeutic outcomes in both *in vitro* and *in vivo* models.

## 2 Materials and Methods

### 2.1 Materials

ATO and PCPDTBT (purity 97% by HPLC) were procured from Yuanye Biotechnology Co., Ltd. (Shanghai, China, sc-53607 and sc-53465). A549 cell-specific culture medium Dulbecco’s Modified Eagle’s Medium (DMEM) (Gibco, Thermo Fisher Scientific, Cat. No. 11965-092, Waltham, MA, USA), phosphate-buffered saline (PBS) (Gibco, Cat. No. 10010-023), trypsin (0.25% trypsin, 1 mM EDTA, with phenol red; Gibco, Cat. No. 25200-056), Fetal Bovine Serum (FBS) (Gibco, Cat. No. 10099-141), Transwell permeable supports with 8.0 μm polycarbonate membrane, and MTT (Cell Proliferation and Cytotoxicity) Assay Kit (MTT; Beyotime Biotechnology, Cat. No. C0009, Shanghai, China) were utilized.

### 2.2 Cell Culture

A549 non-small cell lung cancer cell line was obtained from Procell Life Science & Technology Co., Ltd. (Cat. No. CL-0021, Wuhan, China) with the short tandem repeat (STR) profiling; the cells were confirmed to be free of mycoplasma contamination. Cells were cultured in a DMEM medium at 37°C with 5% CO_2_ and observed for growth status over time in an incubator. The medium was changed daily, and upon reaching over 80% confluency, cells were digested using trypsin. Cells were passaged every two to three days.

### 2.3 Characterization

UV-Vis-NIR spectra were acquired using a NanoDrop 2000c spectrophotometer with samples normalized to equivalent PCPDTBT concentrations (10 μg/mL). Thermal imaging was performed using a Flir E40 compact infrared (IR) thermal imaging camera (FLIR Systems, USA). *In vivo* imaging was conducted using the NightOWL II LB983 instrument (Berthold, Germany). Hydrodynamic size and polydispersity index (PDI) were measured using dynamic light scattering (DLS; Malvern Zetasizer Nano ZS) in PBS (pH 7.4) at 37°C. The DLS using the standard backscattering configuration (scattering angle 173°) at 25°C. Before measurement, samples were diluted with deionized water to a final concentration of 0.2 mg/mL, and passed through a 0.22 μm syringe filter (polyethersulfone (PES); Millipore) to remove dust and large aggregates. Stability was assessed over 5 days with samples stored at 25°C, and data represent mean ± SD of three independent experiments. The photothermal conversion efficiency η was calculated based on previous reports [23].

### 2.4 Synthesis of ATO + PCPDTBT@NPs

**Figure 1:**
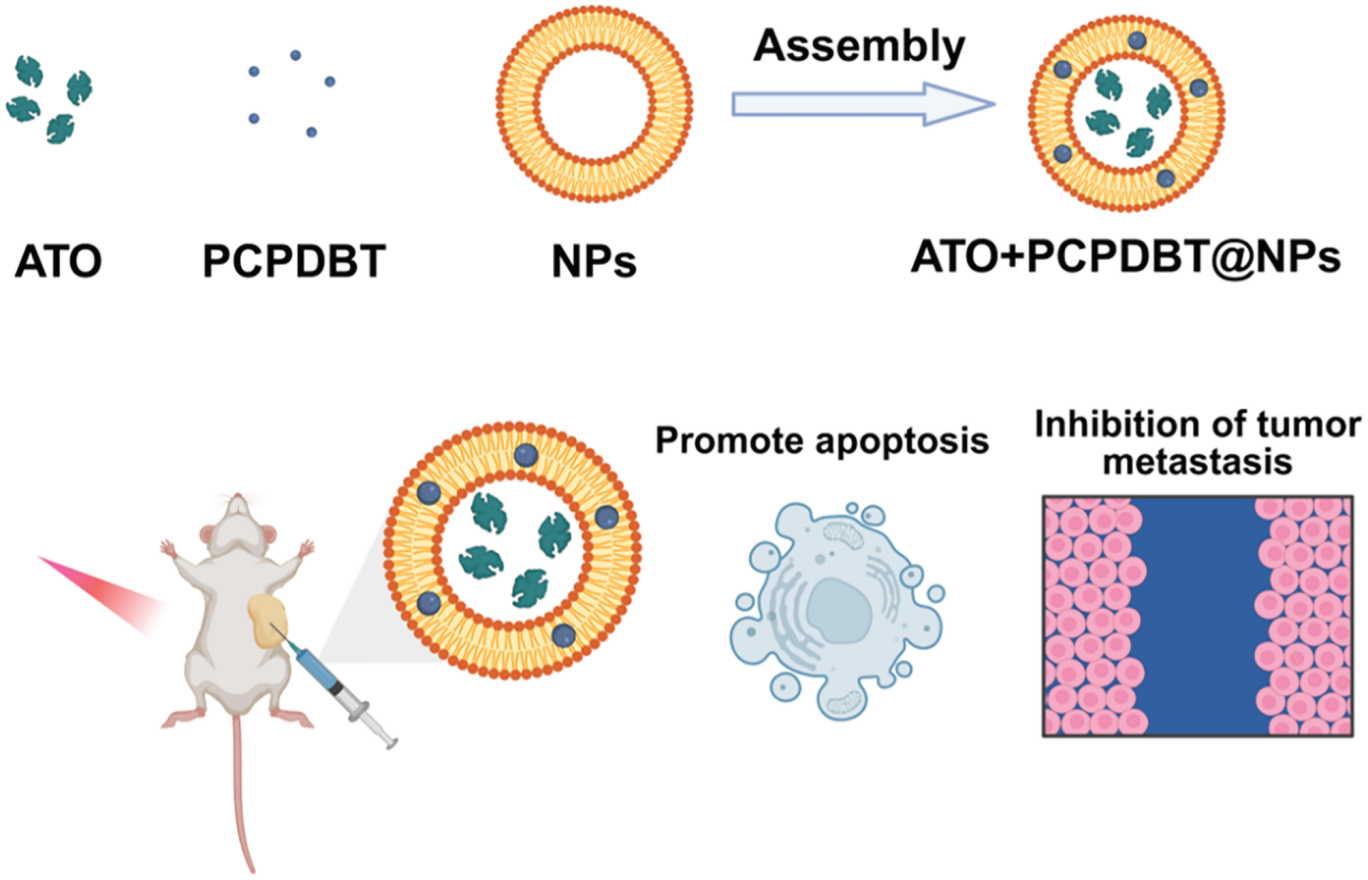
The preparation process of ATO+ PCPDBT@NPs and its schematic diagram as an integrated platform for lung cancer diagnosis and treatment. The nanoparticles are self-assembled by encapsulating Arsenic Trioxide (ATO) and the semiconducting polymer PCPDTBT into liposomes (L-α-phosphatidylcholine/cholesterol) via thin-film hydration, electroporation, and sonication. Upon intravenous administration and 808 nm NIR irradiation, the nanoplatform generates hyperthermia and releases ATO, synergistically promoting tumor cell apoptosis and inhibiting lung cancer metastasis.

As shown in Fig. 1, ATO was dissolved in dimethyl sulfoxide (DMSO) to form a stock solution (10 μmol/L), filtered with a 0.2 μm syringe filter to remove any insoluble compounds, and stored in the dark at −20°C. A mixture containing ATO, PCPDTBT, and L-α-phosphatidylcholine/cholesterol (75 mg:25 mg) dissolved in 1 mL anhydrous ethanol was added to a round-bottom flask containing 10 mL chloroform. The mixture was slowly evaporated on a rotary evaporator (Rotavapor® R-215; Büchi Labortechnik AG, Flawil, Switzerland) to form a uniform film. Subsequently, 10 mL chloroform and 3 mL PBS were added to the round-bottom flask, followed by electroporation and sonication (5 min, 37°C) to form a stable oil-in-water (O/W) phase. The suspension of liposomes was prepared stably using a particle vacuum rotary evaporator (Rotavapor® R-215; Büchi Labortechnik AG) at 40°C, followed by the addition of 7 mL PBS and continuous washing until the suspended liposomes were harvested. Finally, the shape of the nanoliposomes was observed using transmission electron microscopy. NP concentration was quantified gravimetrically after lyophilization and resuspension in PBS, with values normalized to the initial lipid input. If necessary, NPs were labeled with FITC by incubating with fluorescein isothiocyanate (1 mg/mL in DMSO) at 25°C for 2 h, followed by dialysis against PBS to remove unbound dye.

### 2.5 Near-Infrared Laser-Induced Thermal

Conversion Photothermal performance testing of ATO + PCPDTBT@NPs: Different concentrations of ATO + PCPDTBT@NPs were applied under the same power (2 W/cm^2^). A certain amount of ATO + PCPDTBT@NPs was dispersed in water to form different concentrations of 0, 25, 50, and 100 μg/mL. 0.5 mL of each was added to centrifuge tubes, and under a power density of 2 W/cm^2^, irradiated with 808 nm near-infrared laser (FLIR Systems Inc., Wilsonville, OR, USA) for 5 min. Real-time monitoring (FLIR Systems Inc., Wilsonville, OR, USA) was conducted using an infrared thermal imager, recording the highest temperature of the tube every 1 min.

### 2.6 Drug Loading and In Vitro Release Experiments

For drug loading assessment, 2.5 mg of ATO was incubated with 10 mg of NPs in 10 mL PBS (pH 7.4) at 37°C for 24 h to achieve equilibrium. Unencapsulated ATO was removed by centrifugation (500× *g*, 10 min), and the pellets were washed thrice with PBS. The entrapped ATO concentration was quantified by UV-Vis spectrophotometry at 482 nm. *In vitro* release kinetics were evaluated by dispersing 10 mg of the loaded nanoparticles in 10 mL of buffer solutions with distinct pH values (pH 4.7, 7.4, and 9.5) to simulate tumor, physiological, and basic environments, respectively. Following agitation and sonication, the concentration of released ATO in the supernatant was measured at 482 nm (Synergy H1, Agilent BioTek, Winooski, VT, USA).

### 2.7 In Vitro Cytotoxicity

A549 cells were plated in 96-well plates (1 × 10^4^ cells/well) and incubated for 24 h. Following treatment with a concentration gradient (0.1, 1, 10, 100, 400 μg/mL) of ATO/PCPDTBT@NPs for 24 h, cell viability was evaluated using the MTT assay. After treatment, 10 μL MTT solution (5 mg/mL; Beyotime, C0009) was added to each well (final concentration 0.5 mg/mL) and incubated for 4 h at 37°C. The medium was removed and the formazan crystals were dissolved in 150 μL DMSO with gentle shaking for 10 min. Absorbance was measured at 570 nm (reference 630 nm) using a microplate reader (Synergy H1, Agilent BioTek, Winooski, VT, USA). Cell viability percentages were calculated relative to controls, and the half-maximal inhibitory concentration (IC_50_) was derived via non-linear regression using GraphPad Prism software 8.0 (GraphPad Software, LLC, San Diego, CA, USA).

### 2.8 In Vitro Fluorescence Staining

A549 cells (2 × 10^5^/well) were cultured in 12-well chamber slides for 24 h prior to treatment. Following a 24-h incubation with nanoparticles, cells were washed to remove free particles and replenished with fresh medium. For photothermal studies, cells were stained with propidium iodide (PI) (5 μg/mL; Beyotime, C1056) for 5 min and exposed to an 808 nm laser (2 W/cm^2^) for 5 min. To evaluate the combined chemo-photothermal effect, cells underwent an additional 24-h incubation post-irradiation. Samples were fixed with 4% formaldehyde and counterstained with Hoechst 33258 for nuclear visualization before fluorescence imaging (IX73, Olympus, Tokyo, Japan).

### 2.9 In Vivo Infrared Thermal Imaging

Mice were anesthetized with isoflurane (induction 3%–4%, maintenance 1.5%–2.5% in oxygen) and intravenously injected with 0.2 mL of ATO/PCPDTBT@NPs (400 μg/mL) or PBS. One hour post-injection, the tumor regions were irradiated with an 808 nm laser (2 W/cm^2^) positioned perpendicular to the site. Irradiation was performed for 5 min, and surface temperatures (FLIR Systems Inc., Wilsonville, OR, USA) were recorded minutely to monitor thermal distribution.

### 2.10 In Vivo Toxicity Evaluation

To assess systemic toxicity, SCID BALB/c nude mice bearing Luc-A549 subcutaneous tumors (approx. 5 mm diameter) were utilized. Histological analysis was performed on major organs (heart, liver, spleen, lung, and kidney) utilizing hematoxylin and eosin (H&E) staining on tissues fixed in 4% paraformaldehyde for 24 h, paraffin-embedded, and sectioned at 4–5 μm, followed by imaging using an Eclipse Ti2 microscope (Nikon, Tokyo, Japan). Additionally, liver, kidney, and heart functions were quantitatively assessed via serum biochemistry markers. Serum (alanine aminotransferase) ALT, blood urea nitrogen (BUN), and creatine kinase (CK) were measured using an automated biochemistry analyzer (Hitachi 7180, Hitachi High-Technologies, Tokyo, Japan) with corresponding assay kits (Nanjing Jiancheng Bioengineering Institute, Nanjing, China; Cat. No. C009-2-1 for ALT, C013-2-1 for BUN, and A032-1-1 for CK).

### 2.11 In Vivo Antitumor Effect

All animal studies have been approved by the Ethics Committee of Huashan Hospital, Fudan University (Permit number: 2024-HSYY-548) and performed in accordance with the ethical standards. Twenty female BALB/c nude mice (5–6 weeks old, 16–20 g) were obtained from BK (Shanghai, China) and housed under specific-pathogen-free conditions (22°C–25°C, 40%–60% humidity, 12 h light/dark cycle) with ad libitum access to food and water. Luc-A549 cells (approximately 4 × 10^6^ cells/mouse) were injected subcutaneously. When the average tumor diameter reached approximately 5 mm, mice were randomly assigned (random-number generator) into four groups (n = 5 per group) to minimize baseline tumor-size differences; investigators were blinded to group allocation during tumor measurement and endpoint analyses. The four groups were defined as follows: (i) PBS (Control), mice received PBS injections without laser irradiation; (ii) PBS + Laser, mice received PBS injections followed by 808 nm laser irradiation; (iii) ATO/PCPDTBT@NPs, mice received ATO/PCPDTBT@NPs injections without laser irradiation; and (iv) ATO/PCPDTBT@NPs + Laser, mice received ATO/PCPDTBT@NPs injections followed by 808 nm laser irradiation. Mice received tail-vein injections of 0.2 mL ATO/PCPDTBT@NPs (400 μg/mL) or PBS on days 0 and 6. For the laser-treatment groups, mice were anesthetized with isoflurane (induction 3%–4%, maintenance 1.5%–2.5% in oxygen) and tumors were irradiated with an 808 nm laser (2 W/cm^2^, 20 min) at a distance of 2 cm; tumor surface temperature was monitored using an infrared thermal camera (FLIR Systems Inc., Wilsonville, OR, USA). Nanoparticle accumulation in tumors was assessed noninvasively through the skin at 1 h post-injection using an *in vivo* imaging system (IVIS Spectrum, PerkinElmer, Waltham, MA, USA). Tumor length and width, as well as body weight, were recorded every 3 days, and tumor volume was calculated as V = (length × width^2^)/2. At study completion, mice were euthanized by CO_2_ inhalation followed by cervical dislocation as a secondary method, and tumors were excised and weighed.

### 2.12 Statistical Analysis

Data are reported as mean ± standard deviation (SD). Intergroup differences were analyzed using one-way analysis of variance (ANOVA). Statistical significance was defined as *p < 0.05, with higher significance levels denoted as **p < 0.01 and ***p < 0.001. GraphPad Prism 8.0 (GraphPad Software, LLC, San Diego, CA, USA) was used for statistical analysis.

## 3 Results

### 3.1 Characterization of Nanoparticles

**Figure 2:**
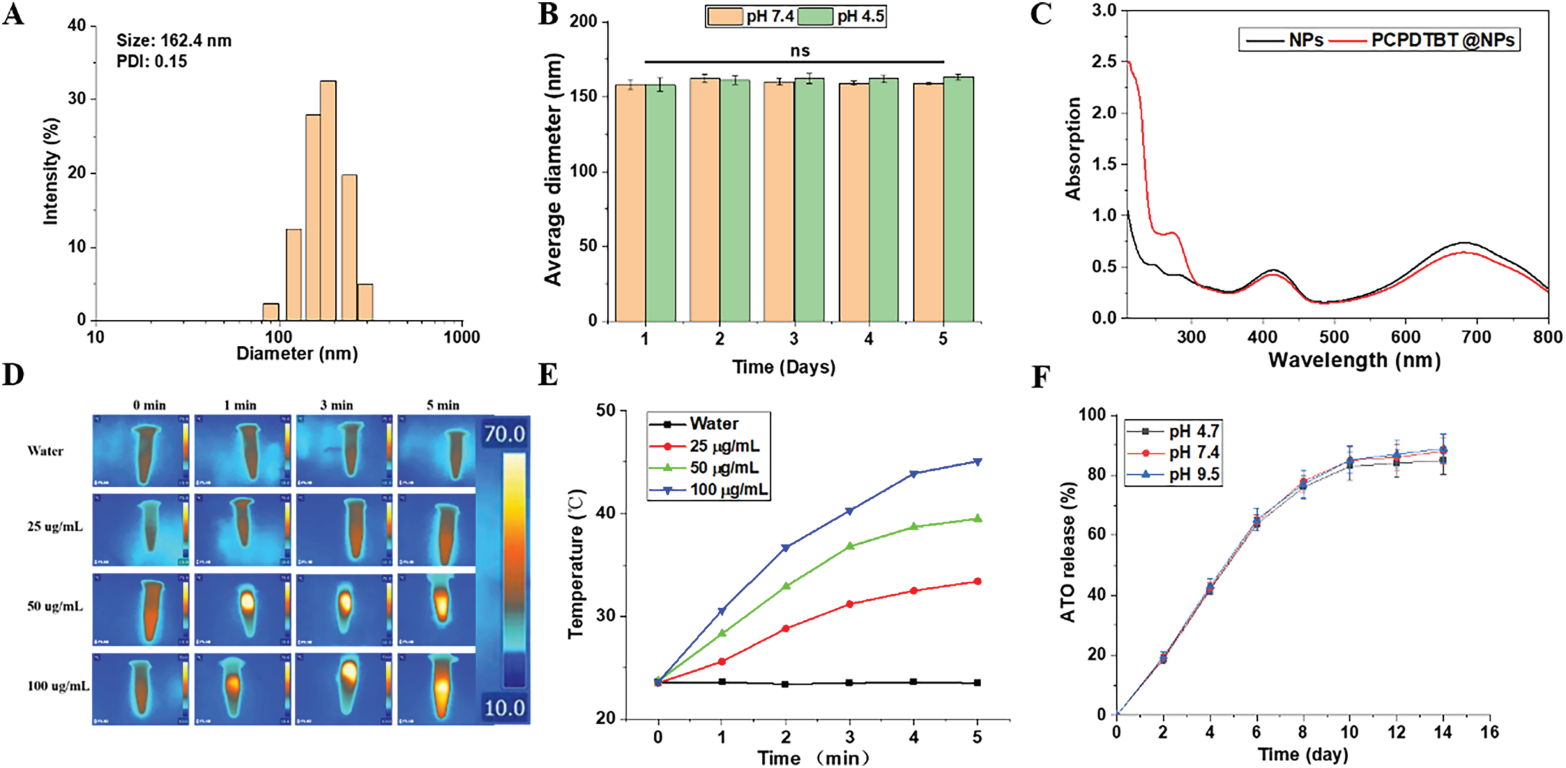
Characterization of ATO + PCPDTBT@NPs. (**A**) Size distribution of ATO + PCPDTBT@NPs as determined by DLS. (**B**) Stability of NPs monitored by DLS over 5 days. (**C**) UV-Vis spectra of NPs before and after loading PCPDTBT. (**D**) Thermal imaging of PCPDTBT with different concentrations at different time points. (**E**) Photothermal heating curves of the PCPDTBT solution with different concentrations under 808 nm NIR irradiation. (**F**) Release rate of ATO over time. ns: no significant difference. ns no significance.

**Figure A1:**
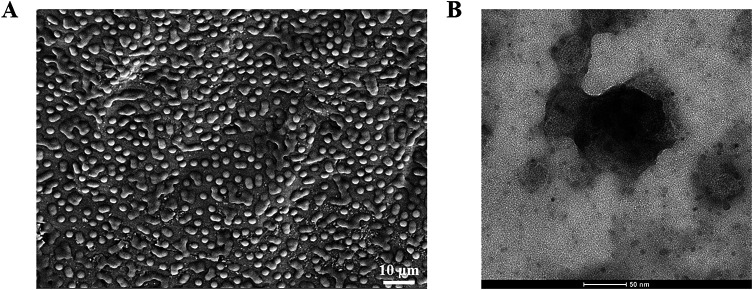
The characterization of ATO + PCPDTBT@NPs. (**A**) The SEM of ATO + PCPDTBT@NPs. (**B**) The TEM of ATO + PCPDTBT@NPs.

**Figure A2:**
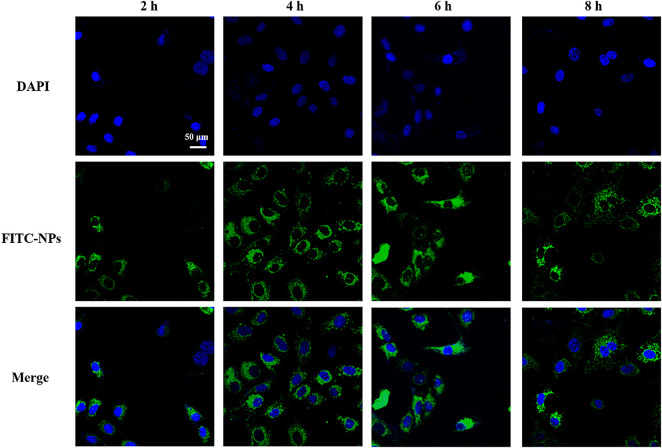
Cellular uptake of ATO + PCPDTBT@NPs. Scale bar, 50 μm.

Dynamic light scattering (DLS) was employed to characterize the synthesized nanoparticles (NPs). As shown in Fig. 2A,B, the hydrodynamic diameter of NPs was approximately 162 nm, displaying a narrow monodisperse size distribution (PDI = 0.15). Furthermore, the stability of the NPs was monitored via DLS during a 5-day storage period at room temperature. As anticipated, no significant changes in size were observed, confirming the sufficient stability of the nano-system (Figs. 2B and A1A,B). Subsequently, we characterized the loading of PCPDTBT through UV-Vis spectroscopy (Fig. 2C). The results showed a distinct absorption peak at 280 nm for PCPDTBT@NPs, indicating the successful loading of PCPDTBT. NIR light (808 nm) provides tissue penetration of 1–2 cm, enabling deep-tumor targeting [24]. As depicted in Fig. 2D,E, under near-infrared laser irradiation, the aqueous solution of ATO + PCPDTBT@NPs exhibited a rapid temperature increase, reaching stability within 5 min. The solution temperature increased with increasing particle concentration. The temperature of the aqueous dispersion rose by 20.8°C within 5 min, while the temperature increase in the control group was less than 2°C under the same level of light intensity, indicating the excellent photothermal conversion efficiency of ATO + PCPDTBT@NPs. The photothermal conversion efficiency η of ATO/PCPDTBT@NPs reached 42.3% under 808 nm irradiation (power density: 2 W/cm^2^), which is higher than most values reported in the literature [25]. We further characterized the sustained release efficiency of ATO, as shown in Fig. 2F, the release efficiency of ATO reached 65.13% within 6 days and then entered a relatively stable curve. The uptake of ATO + PCPDTBT@NPs by cells was analyzed using FITC-labeled NPs. As shown in Fig. A2, with the extension of the incubation time, the fluorescence signal inside the cells gradually increased, and it was mainly localized in the cytoplasmic region. This indicates that ATO + PCPDTBT@NPs were progressively taken up by the cells.

### 3.2 Evaluation of Biocompatibility and Photothermal Synergistic Effect

**Figure 3:**
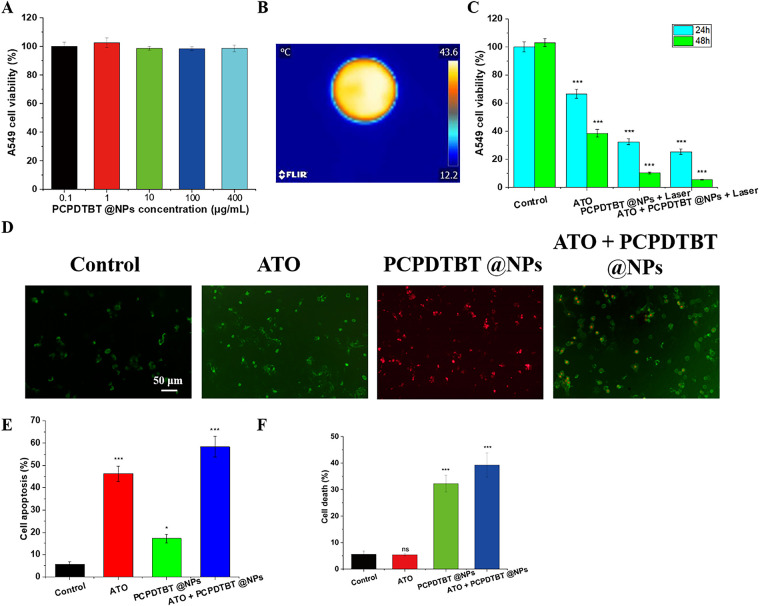
*In vitro* chemotherapy and photothermal synergistic effect of ATO + PCPDTBT@NPs. (**A**) Cytotoxicity of PCPDTBT@NPs particles. (**B**) Photothermal conversion of PCPDTBT@NPs in cell culture dishes. N = 5. (**C**) Synergistic effect of ATO and PCPDTBT@NPs on lung cancer cells A549. (**D**) Representative images of A549 cell apoptosis induced by different treatments. Scale bar, 50 μm. (**E**) Statistical analysis of apoptosis rates in different groups. (**F**) Statistical analysis of necrosis rates in different groups. *p < 0.05, ***p < 0.001. ns: no significant difference.

The biocompatibility of PCPDTBT@NPs nanoparticles was evaluated. As shown in Fig. 3A, there was no significant difference in cell viability after the addition of PCPDTBT@NPs, even at a high concentration of 400 μg/mL. IC_50_ for ATO alone was 12.5 μM, while for ATO/PCPDTBT@NPs with NIR, it was 5.2 μM, demonstrating synergy. Due to the excellent photothermal conversion efficiency of PCPDTBT, as depicted in Fig. 3B, the temperature of a 35 mm culture dish increased to 43°C within 1 min, providing conditions for subsequent photothermal therapy. Furthermore, we tested the *in vitro* photothermal combined effect of ATO + PCPDTBT@NPs. As illustrated in Fig. 3C, the cell viability significantly decreased at 24 h and 48 h after treatment with either ATO or PCPDTBT@NPs alone. Specifically, the cell viability in the ATO and PCPDTBT@NPs combined treatment group decreased to 28.39% at 24 h and 8.6% at 48 h, indicating effective *in vitro* tumor cell killing by chemotherapy and photothermal combined effect. Previous literature has reported that ATO can induce apoptosis of tumor cells. To further validate whether ATO can induce apoptosis of lung cancer cells A549, we co-cultured the cells with various materials. As shown in Fig. 3D–F, after 12 h of culture, early apoptosis and PI staining of cells were performed. The green fluorescence in the ATO group increased significantly, and the apoptosis rate in the co-culture group of ATO + PCPDTBT@NPs further increased, indicating that ATO can promote apoptosis of lung cancer cells A549.

### 3.3 Evaluation of the Effect of ATO + PCPDTBT@NPs Chemotherapy and Photothermal Synergy on Invasion and Metastasis of Lung Cancer Cells

**Figure 4:**
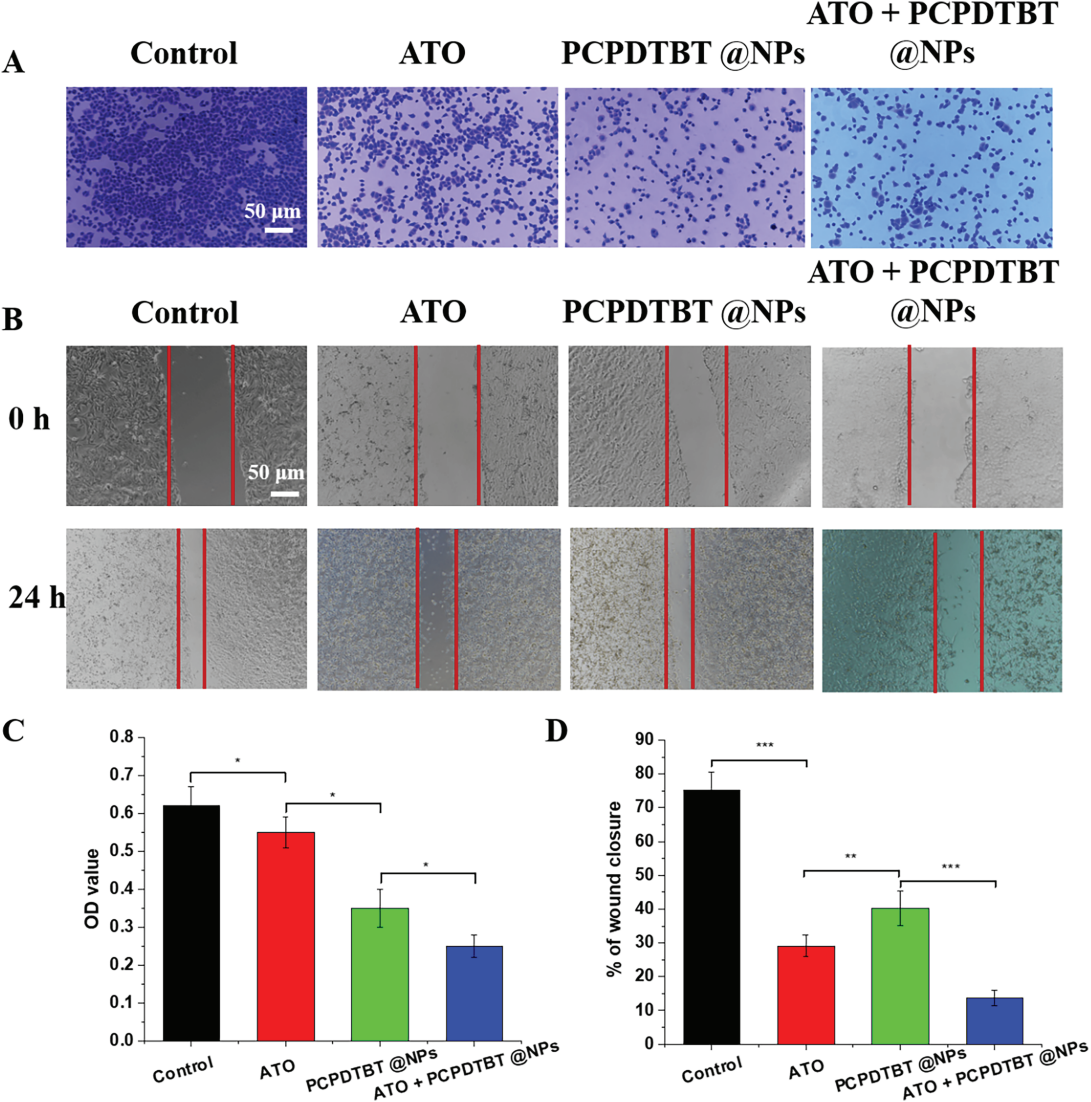
*In vitro* inhibition of A549 cell invasion and migration by ATO + PCPDTBT@NPs. (**A**) Cell invasion assay. (**B**) Cell migration assay. (**C**) Statistical analysis of cell invasion in different groups. N = 5. (**D**) Statistical analysis of cell migration in different groups. N = 5. *p < 0.05, **p < 0.01, ***p < 0.001. Scale bar, 50 μm.

Late-stage metastasis of lung cancer is a major reason for its difficult treatment. We evaluated the effect of ATO + PCPDTBT@NPs chemotherapy and photothermal synergy on the invasion and migration of lung cancer cells (Fig. 4A,B). As shown in Fig. 4C, after the addition of ATO and PCPDTBT@NPs, the invasion and migration of A549 cells were significantly inhibited. Previous experiments have demonstrated that ATO affects invasion and migration by inducing cell apoptosis, while photothermal therapy affects invasion and migration by directly killing tumor cells. As depicted in Fig. 4D, the invasion of cells in the combination treatment group was the lowest, with an OD value of 0.23 compared to 0.62 in the control group. Meanwhile, the cell migration rate was 13.6% in the combination treatment group compared to 74.9% in the control group, indicating that the combined treatment of ATO and PCPDTBT@NPs can significantly inhibit the invasion and migration of lung cancer cells A549 *in vitro*.

### 3.4 In Vivo Biocompatibility of ATO + PCPDTBT@NPs.

**Figure 5:**
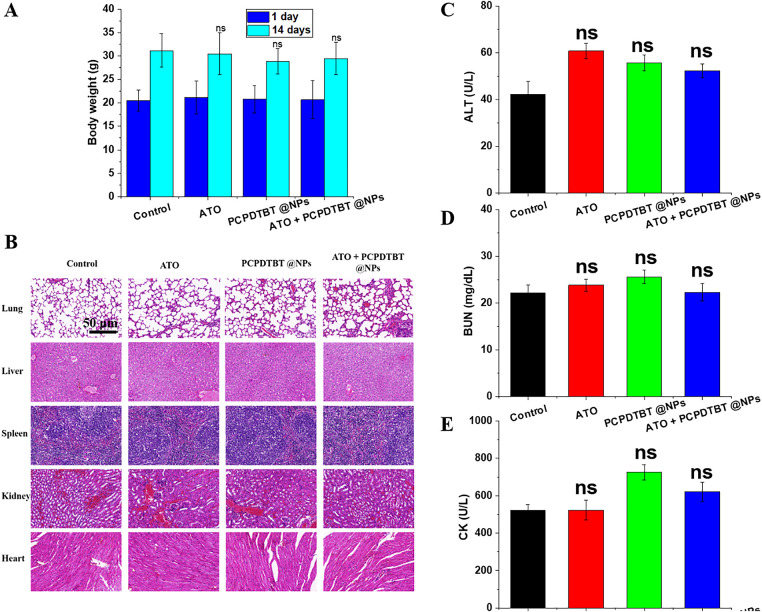
*In vivo* biocompatibility of ATO + PCPDTBT@NPs. (**A**) Changes in body weight of mice in different treatment groups. (**B**) Hematoxylin and eosin (H&E) staining of major organs of mice in different treatment groups. (**C**) Hematological analysis of major organ function toxicity in mice. (**C**) Alanine aminotransferase (ALT): liver function indicator; (**D**) blood urea nitrogen (BUN): kidney function indicator; (**E**) creatine kinase (CK): heart function indicator. Scale bar, 50 μm. ns, no significant difference.

**Figure A3:**
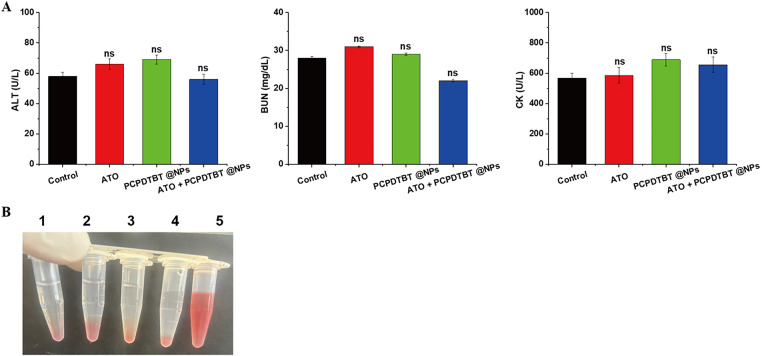
Biosafety assessment. (**A**) Hematological analysis of major organ function toxicity in mice after 30 days. (**B**) Hemolysis assay. (1: Control, 2: ATO, 3: PCPDTBT@NPs, 4: ATO + PCPDTBT@NPs, 5:1% Triton X-100). ns, no significant difference.

The *in vivo* biocompatibility of the nanomaterials represents a critical determinant of their therapeutic applicability and safety. To evaluate systemic tolerance, various nanoparticle formulations were intravenously administered to healthy mice, and body weight was monitored as a general indicator of physiological stress. As illustrated in Figs. 5A and A3A,B, no significant changes in body weight were observed across treatment groups over 14 and 30 days post-injection, suggesting minimal systemic toxicity. Additionally, we evaluated the H&E staining of the heart, liver, spleen, lungs, and kidneys of mice after 14 days of injection, and no significant difference was observed (Fig. 5B), confirming the favorable histocompatibility of the nanoparticle system. Furthermore, we tested the hematology of the mice, and indicators such as ALT, BUN, and CK (Fig. 5C–E) were within the normal fluctuation range, further validating the excellent biocompatibility of the ATO + PCPDTBT@NPs nanoparticles.

### 3.5 In Vivo Inhibition of Tumor Growth and Metastasis by ATO + PCPDTBT@NPs

**Figure 6:**
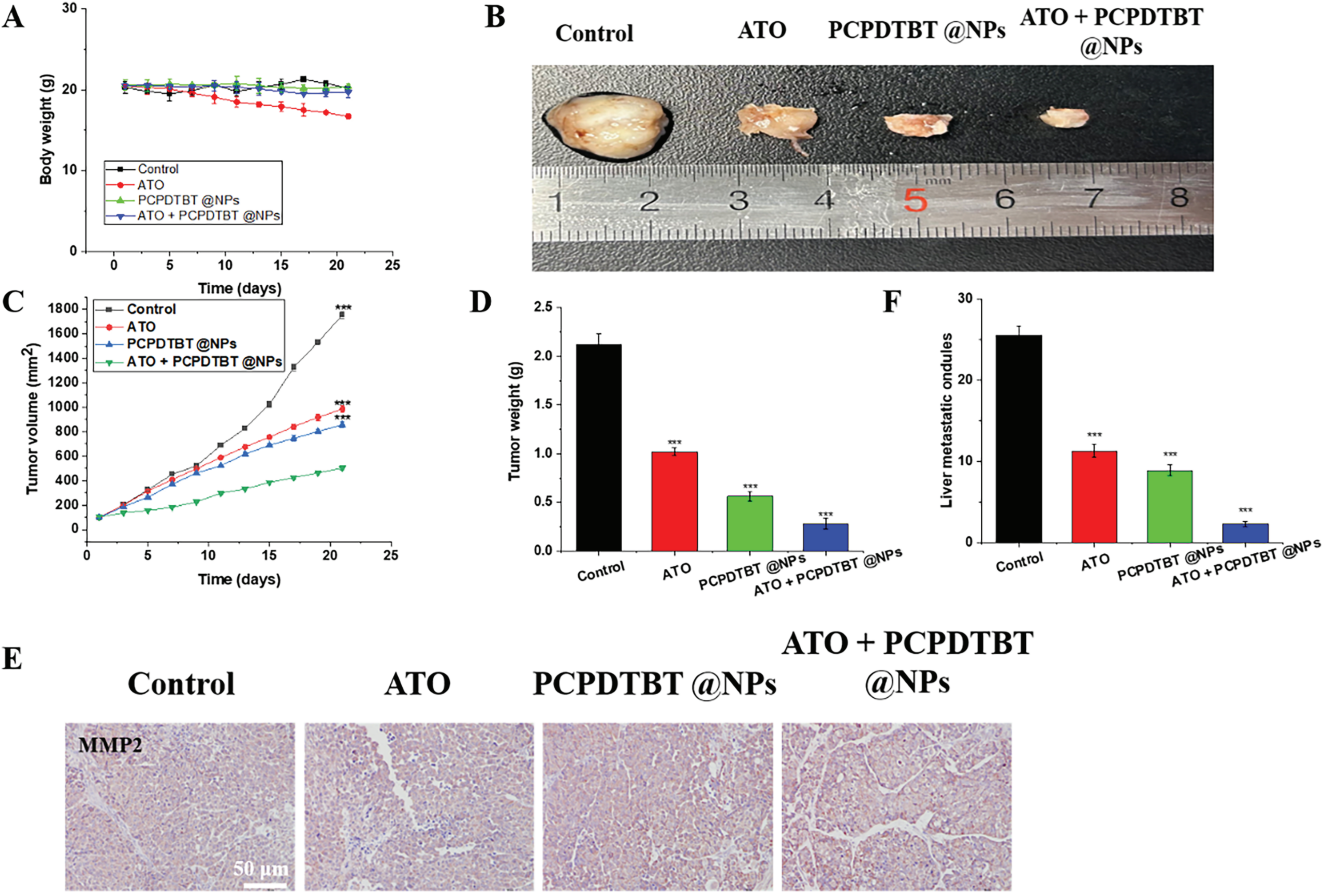
*In vivo* inhibition of tumor growth and metastasis by ATO + PCPDTBT@NPs. (**A**) Changes in body weight of mice in each group during treatment. (**B**) Macroscopic images of tumors from mice in each group. (**C**) Changes in tumor volume in each group. (**D**) Changes in the weight of mice in each group. (**E**) MMP2 staining of tumors. (**F**) Statistical analysis of hepatic nodules. N = 5. ***p < 0.001. Scale bar, 50 μm.

**Figure A4:**
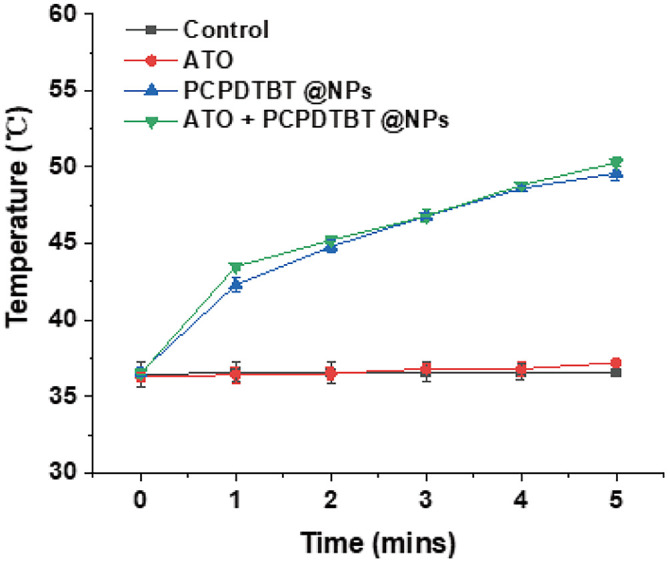
The temperature of the nano-material in mice after NIR.

**Figure A5:**
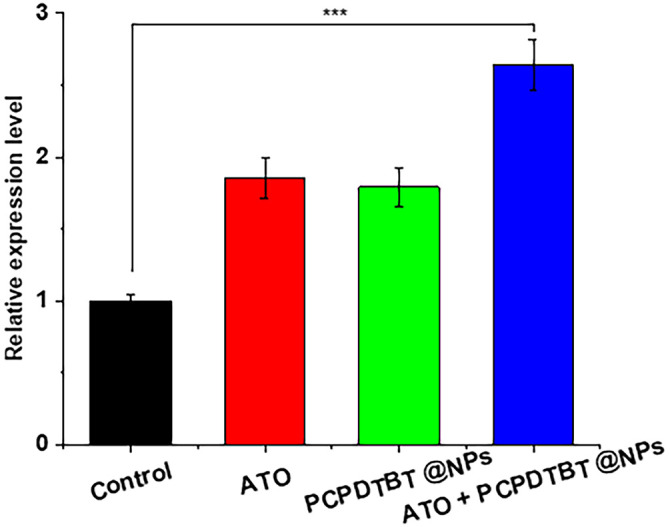
Quantitative analysis of apoptosis-related marker Caspase-3 in tumor tissues. ***p < 0.001.

**Figure A6:**
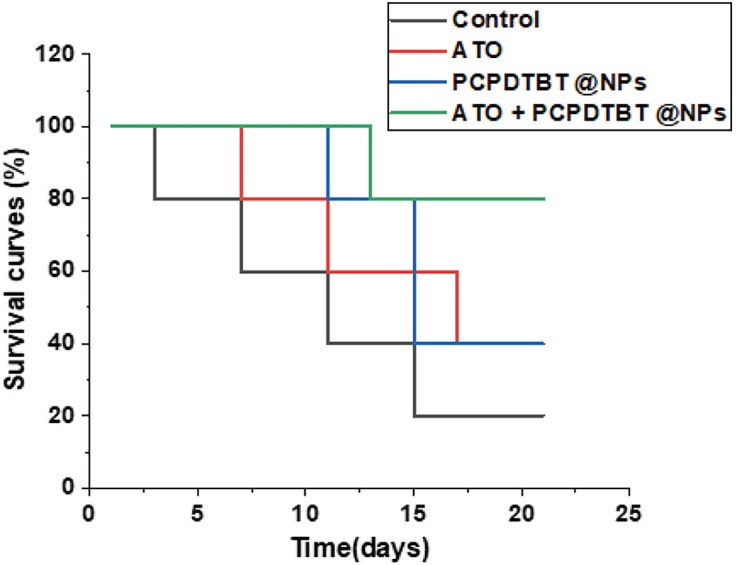
The survival curves of each group.

The antitumor efficiency of NPs was evaluated on Balb/c mice carrying subcutaneous tumors. As shown in Fig. 6A, the average body weight of mice treated with ATO decreased by approximately 3 g, indicating the toxicity of ATO to tumor-bearing mice. However, the reduction in body weight of mice in other groups was not negligible, further confirming that functionalized NPs as drug carriers can effectively reduce the adverse reactions of ATO in mice. This is attributed to the combined effect of ATO + PCPDTBT@NPs photothermal and chemotherapy (Fig. A4). Images and weights of tumors after euthanization of mice also confirmed the different treatment effects on tumor growth (Fig. 6B–D). Meanwhile, at the end of the treatment, the tumor volume of mice injected with ATO and NPs alone decreased by 2.5 and 3 times, respectively (Fig. 6C). To further investigate the molecular mechanism of cell death induced by the synergistic therapy, we performed a quantitative analysis of Caspase-3, a key executioner of apoptosis. As presented in Fig. A5, the expression level of Caspase-3 was significantly elevated in the ATO/PCPDTBT@NPs + Laser group compared to the control and monotherapy groups. This result confirms that the photothermal-chemotherapy combination effectively triggers programmed cell death in tumor tissues, which aligns with the observed reduction in tumor volume. Furthermore, the combination of both showed further suppression of tumor volume and survival rate (Fig. A6). Consistent with the changes in tumor growth, the smallest tumor masses were observed in mice treated with combined therapy. Moreover, a high concentration of cytoplasm with abundant vesicles and significant necrosis was observed in the combination treatment group, indicating that ATO + PCPDTBT@NPs could effectively inhibit tumor occurrence and exhibit excellent therapeutic effects *in vivo*. Additionally, we analyzed the mechanism of inhibition of tumor metastasis by ATO + PCPDTBT@NPs through immunohistochemical analysis (Fig. 6E,F). MMP2 was downregulated in the combined treatment group, further confirming the satisfactory therapeutic effect of combined treatment.

**Figure A7:**
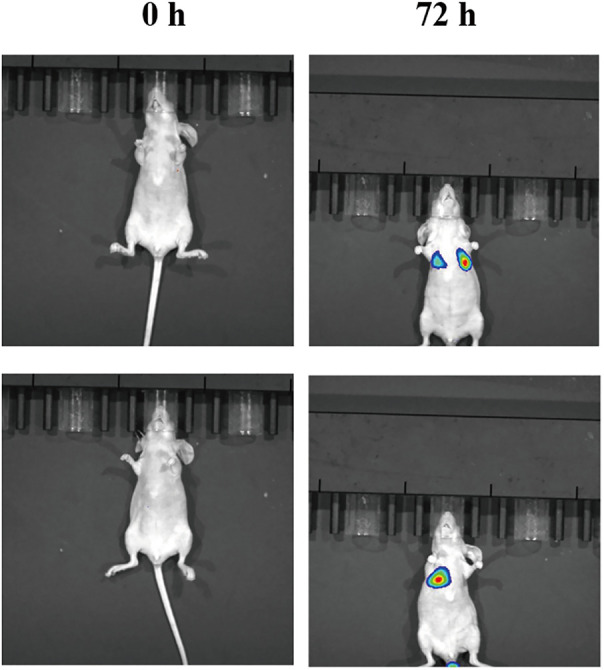
Representative images of ATO + PCPDTBT@NPs for lung cancer diagnosis.

**Figure A8:**
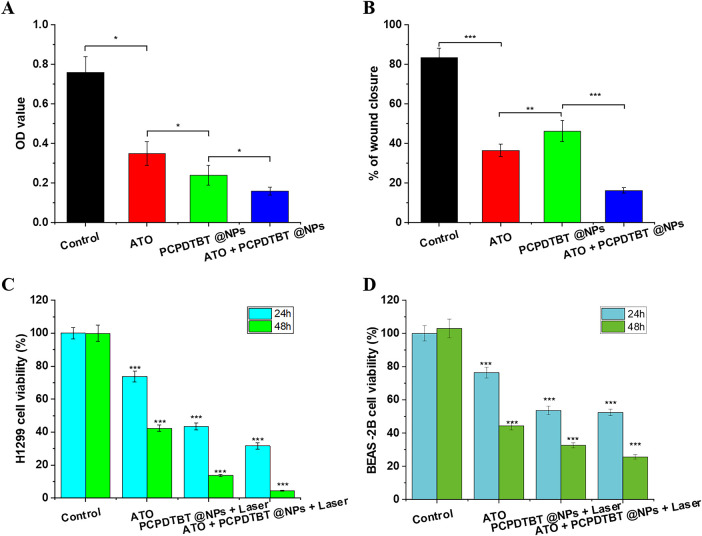
*In vitro* validation of synergistic efficacy and safety profile across cell lines. (**A**) Viability of H1299 cells treated with control, free ATO (5 μM), PCPDTBT@NPs (100 μg/mL), or ATO/PCPDTBT@NPs (5 μM ATO + 100 μg/mL NPs) for 24/48 h. (**B**) Wound closure rates in H1299 monolayers after 24 h treatment. (**C**) Viability of H1299 NSCLC cells (p53-null) post-treatment with/without 808 nm laser (2 W/cm^2^, 5 min). Note enhanced cytotoxicity in the combination group. (**D**) BEAS-2B normal lung epithelial cells exhibit significantly higher resistance to all treatments than cancer cells, confirming tumor-selective toxicity. *p < 0.05, **p < 0.01, ***p < 0.001.

In parallel, our study also elucidated the diagnostic potential of ATO/PCPDTBT@NPs in the context of lung cancer. Advanced-stage lung cancer is frequently characterized by metastasis, which significantly complicates both therapeutic intervention and early detection. As illustrated in Fig. A7, the application of external near-infrared laser irradiation enabled clear visualization of primary tumor sites and metastatic lesions. This real-time optical feedback is of considerable clinical value, offering a dual-function theranostic platform that not only facilitates targeted treatment but also enhances tumor detection and monitoring. To further confirm the biosafety and therapeutic selectivity of the nanoplatform, the cytotoxicity of ATO/PCPDTBT@NPs was evaluated on normal human lung epithelial cells (BEAS-2B) using the MTT assay. As illustrated in Fig. A8A–D, the cell viability of normal cells remained above 90% even when the concentration of nanoparticles reached 100 μg/mL. This stands in sharp contrast to the significant dose-dependent cytotoxicity observed in A549 lung cancer cells, confirming that the developed nanoplatform possesses excellent biocompatibility and high selectivity, effectively minimizing potential damage to healthy tissues.

## 4 Discussion

In this study, we developed nanovesicles co-loaded with arsenic trioxide (ATO) and the semiconducting polymer PCPDTBT (ATO/PCPDTBT@NPs) as a metal-free platform for synergistic chemo–photothermal therapy against non-small cell lung cancer. The nanoparticles exhibited a uniform nanoscale size, good colloidal stability, efficient 808 nm photothermal conversion, and sustained ATO release, while maintaining excellent hemocompatibility and low cytotoxicity toward normal cells. These properties indicate that encapsulating PCPDTBT within liposomal nanovesicles is an effective strategy to obtain robust photothermal performance without relying on inorganic photothermal agents [26].

Functionally, ATO/PCPDTBT@NPs under NIR irradiation markedly enhanced antitumor efficacy compared with free ATO or photothermal treatment alone. The combination treatment significantly reduced A549 cell viability, increased apoptosis, and inhibited cell migration and invasion, suggesting a clear chemo–photothermal synergy rather than a simple additive effect [27]. *In vivo*, ATO/PCPDTBT@NPs plus NIR irradiation achieved superior tumor growth inhibition and downregulation of metastasis-related markers, while body weight, hematological indices, and major organ histology remained largely within normal ranges [28]. These findings indicate that nanoparticle-based delivery can improve the therapeutic index of ATO by enhancing tumor-specific effects and reducing systemic toxicity.

In addition to therapy, the strong NIR absorption of PCPDTBT enabled photothermal imaging and fluorescence visualization of tumors, highlighting the theranostic potential of this platform for simultaneous imaging and treatment [29]. Nonetheless, several limitations should be acknowledged. The mechanistic basis of the observed synergy was not fully dissected, and the current data are derived from subcutaneous xenograft models that do not completely recapitulate the clinical behavior of lung cancer. Moreover, long-term biodistribution, clearance, and immunogenicity of ATO/PCPDTBT@NPs remain to be systematically evaluated.

Overall, our results provide proof-of-concept that ATO/PCPDTBT@NPs offer a promising, biocompatible nanoplatform that integrates photothermal ablation, chemotherapy, metastasis inhibition, and imaging for lung cancer management. Future work should focus on detailed mechanistic studies, orthotopic or metastatic models, and comprehensive safety evaluation to further advance this semiconducting polymer–based theranostic system toward potential clinical translation.

## 5 Conclusion

In summary, a facile self-assembly synthesis strategy was employed to fabricate nanovesicles co-loaded with ATO and PCPDTBT, designed for integrated photochemotherapy and diagnostic applications in lung cancer. The resulting ATO/PCPDTBT@NPs demonstrated excellent hemocompatibility and markedly reduced cytotoxicity toward human lung carcinoma A549 cells. The semiconducting polymer PCPDTBT exhibited superior photothermal conversion efficiency, rapidly elevating local tumor temperatures upon near-infrared laser irradiation. Concurrently, ATO promoted apoptosis, synergistically enhancing the nanomedicine’s overall therapeutic efficacy. *In vitro* investigations confirmed efficient cellular internalization and potent photothermal cytotoxicity following laser exposure. *In vivo* studies revealed that the nanoparticles preferentially accumulated at tumor sites and facilitated tumor detection via NIR fluorescence. Moreover, the combined photothermal and chemotherapeutic intervention significantly suppressed tumor progression, underscoring the translational potential of ATO/PCPDTBT@NPs as a promising theranostic platform for lung cancer management.

## Data Availability

Data for this article are either included in the manuscript and the supporting information or are available upon request.
